# International workshop on insecticide resistance in vectors of arboviruses, December 2016, Rio de Janeiro, Brazil

**DOI:** 10.1186/s13071-017-2224-3

**Published:** 2017-06-02

**Authors:** Vincent Corbel, Dina M. Fonseca, David Weetman, João Pinto, Nicole L. Achee, Fabrice Chandre, Mamadou B. Coulibaly, Isabelle Dusfour, John Grieco, Waraporn Juntarajumnong, Audrey Lenhart, Ademir J. Martins, Catherine Moyes, Lee Ching Ng, Kamaraju Raghavendra, Hassan Vatandoost, John Vontas, Pie Muller, Shinji Kasai, Florence Fouque, Raman Velayudhan, Claire Durot, Jean-Philippe David

**Affiliations:** 1 Institut de Recherche pour le Développement (IRD), Maladies Infectieuses et Vecteurs, Ecologie, Génétique, Evolution et Contrôle (MIVEGEC, UM1-CNRS 5290-IRD 224), B.P. 64501, 911 Avenue Agropolis, 34394, Cedex 5 Montpellier, France; 2 Rutgers University (RU), Center for Vector Biology, 180 Jones Avenue, New Brunswick, NJ 08901 USA; 3 Liverpool School of Tropical Medicine (LSTM), Department of Vector Biology, Pembroke Place, Liverpool, L35QA UK; 4 Global Health and Tropical Medicine, GHTM, Instituto de Higiene e Medicina Tropical, IHMT, Universidade Nova de Lisboa, UNL, Rua da Junqueira 100, 1349–008 Lisbon, Portugal; 5 Department of Biological Sciences, University of Notre Dame (UND), Eck Institute for Global Health, 239 Galvin Life Science Center, Notre Dame, Indiana, 46556 USA; 6 Malaria Research and Training Center (MRTC), Point G, Bamako, B.P 1805 Mali; 7 Institut Pasteur de la Guyane (IPG), 23 avenue Pasteur B.P. 6010, 97306, Cedex Cayenne, French Guiana; 8 Department of Entomology, Kasetsart University (KU), 50 Ngam Wong Wan Rd, Ladyaow, Bangkok, Chatuchak 10900 Thailand; 9 Center for Global Health/Division of Parasitic Diseases and Malaria/Entomology Branch, U.S. Centers for Disease Control and Prevention (CDC), 1600 Clifton Rd. NE, MS G-49; Bldg. 23, Atlanta, GA 30329 USA; 10 Instituto Oswaldo Cruz (Fiocruz), Avenida Brasil 4365, Rio de Janeiro/RJ CEP, Manguinhos 21040–360 Brazil; 11 Big Data Institute, Li Ka Shing Centre for Health Information and Discovery, University of Oxford, Oxford, OX3 7LF UK; 12 Environmental Health Institute (EHI), National Environment Agency (NEA), 11 Biopolis Way, Helios Block, #04-03/04 & #06-05/08, Singapore, Republic of Singapore; 13 National Institute of Malaria Research (NIMR), Department of Health Research, GoI Sector 8, Dwarka, Delhi, 110 077 India; 14 Department of Medical Entomology & Vector Control, School of Public Health and Institute for Environmental Research, Tehran University of Medical Sciences (TUMS), Pour Sina Street, P.O. Box: 14155–6446, Tehran, Iran; 15 Institute Molecular Biology and Biotechnology (IMBB), Foundation for Research and Technology (FORTH), Panepistimioupoli, Voutes, 70013, Heraklio, Crete, Greece; 16 Pesticide Science Laboratory, Agricultural University of Athens, Ieara Odoes 75, 118 Athens, Greece; 17 Department of Epidemiology and Public Health, Swiss Tropical and Public Health Institute, Socinstrasse 57, PO Box 4002, Basel, Switzerland; 18 Department of Medical Entomology, National Institute of Infectious Diseases, 1-23-1 Toyama, Shinjukuku, Tokyo, Japan; 19 Vector Environment and Society Unit, The Special Programme for Research and Training in Tropical Diseases World Health Organization, 20, avenue Appia, CH-1211, 27 Geneva, Switzerland; 20 Vector Ecology and Management, Department of Control of Neglected Tropical Diseases (HTM/NTD), World Health Organization, 20 Avenue Appia, CH-1211, 27 Geneva, Switzerland; 21 Centre National de la Recherche Scientifique (CNRS), Laboratoire d’Ecologie Alpine (LECA), UMR 5553 CNRS Université Grenoble-Alpes, Domaine universitaire de Saint-Martin d’Hères, 2233 rue de la piscine, 38041, Cedex 9 Grenoble, France

**Keywords:** Arbovirus, Mosquito, Insecticide resistance, Vector control, WIN network, Review, Standardization, Strategic planning

## Abstract

Vector-borne diseases transmitted by insect vectors such as mosquitoes occur in over 100 countries and affect almost half of the world’s population. Dengue is currently the most prevalent arboviral disease but chikungunya, Zika and yellow fever show increasing prevalence and severity. Vector control, mainly by the use of insecticides, play a key role in disease prevention but the use of the same chemicals for more than 40 years, together with the dissemination of mosquitoes by trade and environmental changes, resulted in the global spread of insecticide resistance. In this context, innovative tools and strategies for vector control, including the management of resistance, are urgently needed. This report summarizes the main outputs of the first international workshop on Insecticide resistance in vectors of arboviruses held in Rio de Janeiro, Brazil, 5–8 December 2016. The primary aims of this workshop were to identify strategies for the development and implementation of standardized insecticide resistance management, also to allow comparisons across nations and across time, and to define research priorities for control of vectors of arboviruses. The workshop brought together 163 participants from 28 nationalities and was accessible, live, through the web (> 70,000 web-accesses over 3 days).

## Background

Insecticide Resistance is recognized by the World Health Organization (WHO) as an important threat to vector-borne diseases control and prevention. There is an urgent need to identify the countries and regions where resistance could challenge vector control and to accelerate the deployment of innovative tools for vector control. Better understanding of the strength and dynamics of insecticide resistance will help to develop a global strategy for insecticide resistance containment in arboviruses vectors.

In March 2016, TDR, the Special Program for Research and Training in Tropical Diseases in collaboration with the WHO Neglected Tropical Diseases Department (NTD/WHO), has supported the launch of the first-ever international network to track insecticide resistance in mosquito vectors of arboviruses. The Worldwide Insecticide-resistance Network (WIN) https://win-network.ird.fr/), aims to enhance the monitoring of insecticide resistance worldwide, filling knowledge gaps and guiding decision making for improved insecticide resistance management strategies and vector control [1].

In December 5-8th 2016, the first International Workshop on “**Insecticide resistance in vectors of emerging arboviruses: Challenge and prospects for vector control**” was held in Rio de Janeiro, Brazil, and was organized jointly by the Brazilian Instituto Oswaldo Cruz (IOC) and the French Institut de Recherche pour le Développement (IRD) and Centre National de la Recherche Scientifique (CNRS). Representatives from 94 institutions working on vector-borne diseases were present including research institutions and universities, international organizations (WHO, CDC), development agencies (e.g. USAID), ministries of health and members of the private sector. The workshop brought together 163 participants from 28 nationalities and was accessible, live, through the web (> 70,000 web-accesses over the 3 days). The workshop served as a forum to identify priorities in vector research and to provide national authorities with recommendations for the improvement of insecticide resistance management and deployment of alternative vector control tools.

During the workshop, three scientific plenary sessions were organized: the first session dedicated to the **“Emergence of arboviruses diseases”** addressed the causes and consequences of the expansion of arboviral diseases and their vectors and discussed challenges for improving their control. The second session dedicated to **“Insecticide resistance”** focused on the current distribution, mechanisms, fitness cost and impact of insecticide resistance on vector control and discussed challenges in resistance diagnostics and monitoring. The last plenary session dedicated to **“Innovative vector control”** presented new developments in chemical, biological and genetic approaches for controlling mosquito vectors and reducing arboviruses transmission. Each plenary session comprised multiple presentations by scientists followed by open discussions with all participants. Scientific sessions were followed by a plenary **“Industry session”** where representatives of the agrochemical sector and vector control consortium presented innovative tools and promoted private-public partnership for the development of new public health insecticides. In addition, 30 posters were presented by scientists and industry. Finally, two discussion round tables open to all participants were organized to leverage the knowledge of the audience into strategies that may accelerate the translation of vector research into policies and programs. The meeting agenda, list of speakers, registered participants and presentations are available at https://win-network.ird.fr/.

## Welcoming addresses

The first day was opened with welcoming addresses by representatives of the Brazilian Ministry of Health, the French Ministry of Foreign Affairs in Brazil, the Instituto Oswaldo Cruz, the WHO NTD and TDR departments, and the Pan American Health Organization (PAHO). All speakers acknowledged the scale of the emergency caused by arboviral diseases worldwide and called for stronger commitment and international cooperation to sustain vector control and combat insecticide resistance. Dr. Ademir Martins (FIOCRUZ/IOC) welcomed participants and presented the objectives of the meeting. Dr. Vincent Corbel (IRD, France) then introduced the Worldwide Insecticide resistance Network (WIN) [1] that brings together 19 internationally recognized institutions in vector research with the aim to monitor and contain insecticide resistance worldwide. The WIN, which is supported by the WHO-TDR program, was commissioned to develop in-depth reviews of the current knowledge and gaps in insecticide resistance management and effective vector control strategies (accessible online through the WIN website). The ultimate goal of the WIN is to develop an international consortium aiming at strengthening the capacity of national authorities in insecticide resistance monitoring and management.

## Session 1: Emergence of arbovirus diseases

**Fig. 1:**
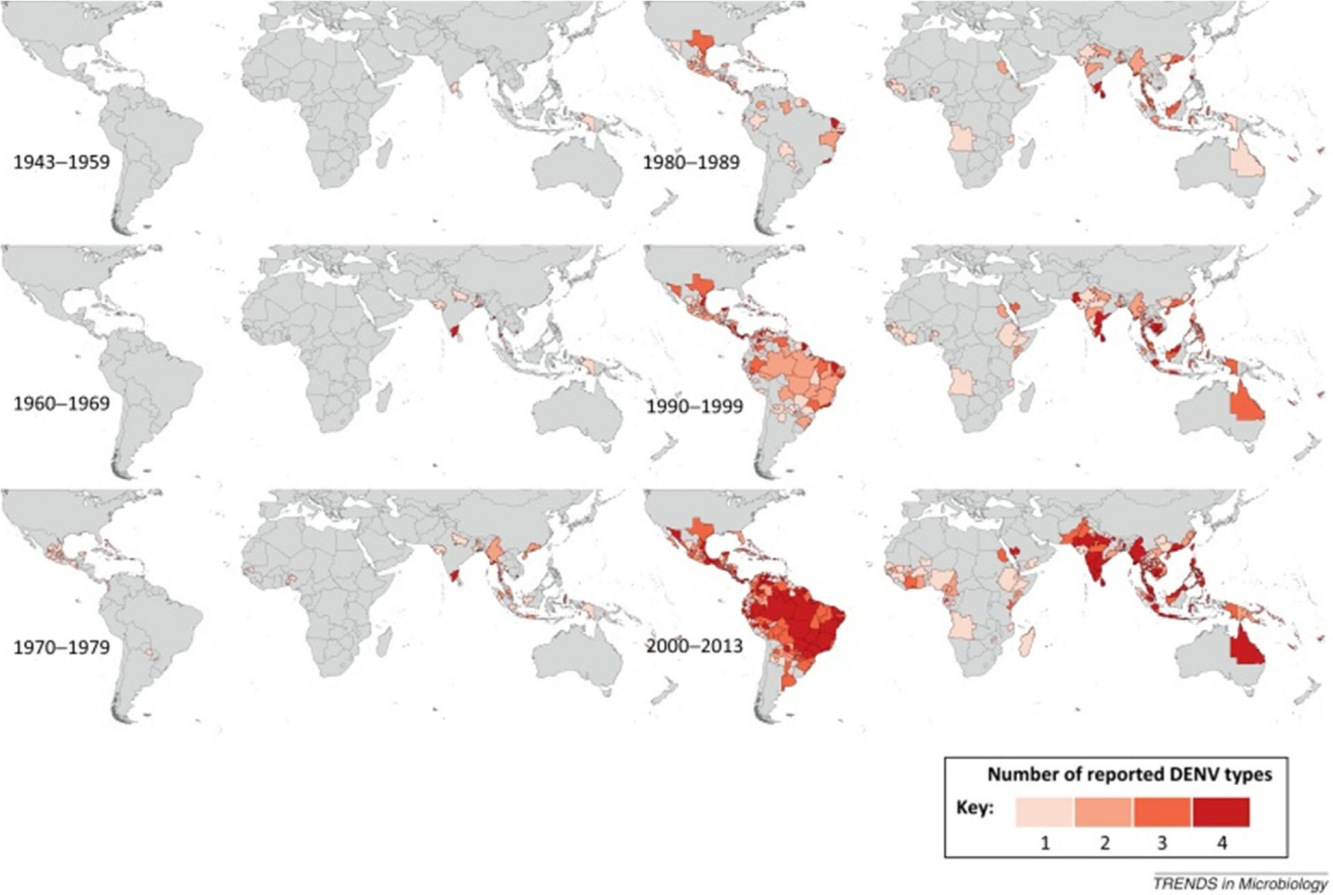
Global spread of dengue virus (Courtesy of Dr. A. Wilder-Smith). Copyright: Creative Commons Attribution 4.0 License (https://creativecommons.org/licenses/by/4.0/). Citation: Messina et al. (2014) Global spread of dengue virus types: mapping the 70 year history. *Trends in Microbiology.* 2014;22(3):138–146 [67]

Prof. Annelies Wilder-Smith (Lee Kong Chian School of Medicine, Singapore) opened the first session by summarizing the increasing problem caused by epidemic arboviral diseases in the twenty-first century. The last five decades have witnessed an unprecedented emergence of epidemics of arboviral diseases including dengue, chikungunya, yellow fever, and Zika transmitted by *Aedes* mosquitoes. The recent epidemiological history of what became the Zika emergency started with the 2007 outbreak in Micronesia, followed by Polynesia in 2013, and Brazil in 2014 from where it rapidly spread to neighboring countries and the Caribbean. Recent outbreaks of yellow fever (YF) began in Angola (884 confirmed cases) and neighboring countries (Mauritania, Kenya, DRC) in December 2015 and the YF virus spread to Asia for the first time in history (11 cases reported in China). Chikungunya virus (CHIKV) caused havoc in 2013–2014 when a reemerging strain was introduced into and swept through the Caribbean and Latin America at an unprecedented speed and scale. However, the most important arboviral disease remains dengue due to its broad distribution, increased epidemic activity, hyper-endemicity and disease severity. The frequency and magnitude of dengue epidemics have increased significantly over the past 40 years (Fig. 1). Overcrowded urban settings facilitate transmission *via* *Aedes* mosquitoes as documented recently in Singapore [2]. Among the major causes of emergence of arboviral diseases are demographic changes, massive urbanization, population movement, trade, and lack of effective control, all of which favor the worldwide spread of both the virus and the vector.

**Fig. 2:**
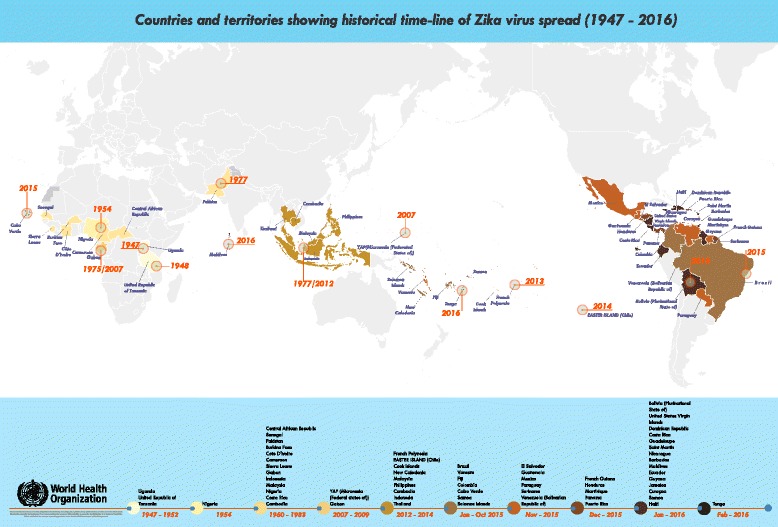
Global spread of Zika virus 1947–2016 (in: WHO June 2016, http://www.who.int/emergencies/zika-virus/zika_timeline.pdf?ua=1)

Dr. Pedro Vasconcelos (Instituto Evandro Chagas, Brazil), described the causes and consequences of the Zika virus outbreak in Brazil. First occurrences of an exanthematic syndrome of Zika were reported in the State of Rio Grande do Norte in October 2014 [3]. Results of phylogenetic and molecular clock-based analyses showed a single introduction of ZIKV into the Americas possibly from French Polynesia [4]. The virus has caused 1.5 million cases in Brazil and may have been introduced during an international sporting event [5]. First associations between Zika and microcephaly were demonstrated in northeastern Brazil [6]. Subsequently 72 countries and territories have reported evidence of mosquito-borne Zika virus transmission since 2007 (Fig. 2), among which 20 countries have reported microcephaly and other usually-rare CNS malformations, especially Guillain-Barré syndrome, potentially associated with Zika virus infection [7]. Multiple modes of transmission of Zika virus are possible: vector, sexual, perinatal, saliva, congenital, blood transfusion, and possibly urine. Association with other malformations and other complications (deafness, ocular alterations, arthrogryposis, cranio-facial size disproportion, calcifications, etc.) is likely. Vaccines for Zika are at early stage of development [8].

Dr. Raman Velayudhan (Neglected Tropical Diseases Department, World Health Organization, Switzerland) described the WHO response and preparedness for arbovirus vector control. The presentation started by reviewing early success stories of eradication campaigns of arboviral diseases in South America using source reduction and effective targeted residual spraying with insecticides as part of centralized, vertically-structured programs. According to the WHO, well-implemented vector control programs using targeted residual spraying, space spraying, larval control and personal protective measures can be effective in reducing the transmission of *Aedes*-borne diseases including Zika. Therefore, sustaining vector control must remain a priority. This includes accurate vector and clinical surveillance, implementation of Integrated Vector Management (IVM) and strengthening national capacity for better preparedness for the emerging threat of arboviruses. The Global Vector Control Response currently being developed by the WHO will aim to enhance vector control capacity and foster research and innovation in this field.

Prof. Dina Fonseca (Rutgers University, USA) gave a talk on insecticide resistance and the globalization of *Aedes* mosquitoes. Invasive mosquitoes such as *Culex pipiens*, *Cx. quinquefasciatus*, *Ae. aegypti* and *Ae. albopictus* have driven most of the recent arboviral epidemics. Invasive mosquitoes are widespread and abundant because they exploit human environments. *Aedes aegypti*, originally from Africa, *Aedes albopictus* as well as more recently *Aedes japonicus*, originally from Asia, have become established worldwide. Population genetic studies have shown that *Ae. aegypti* spread across the New World probably around 1500 CE, *Ae. albopictus* after the 1960s and *Ae. japonicus* in the late 1990s [9, 10] but successive (re-) introductions of these species appear as a common pattern [11]. Anthropization and increases in the worldwide circulation of goods also favor mosquito expansion by creating favorable conditions promoting the dispersal of immature stages [12]. One of the primary threats associated with mosquito expansions is the introduction of insecticide resistant populations into new territories, potentially undermining decades of IR management. Understanding the factors that promote invasiveness will allow the development of better strategies for prevention and control of invasive disease vectors.

Dr. Anne Wilson (Durham University, School of Biosciences, UK) presented the concept of Integrated Vector Management (IVM) in the context of arbovirus control. IVM is an evidence-based, adaptive and multi-sectoral approach to vector control [13]. Briefly, it involves a range of vector control tools used either alone or in combination selected based on knowledge of the local vector ecology and disease epidemiology. Dr. Wilson provided examples of IVM strategies for *Aedes*-control and pinpointed the benefits of IVM for insecticide resistance management. In Mexico, improving housing by using long lasting insecticide-treated house screens and targeted larviciding resulted in long lasting significant reductions in *Ae. aegypti* infestations [14]. Cluster randomized controlled trials in Nicaragua and Mexico demonstrated that evidence-based community mobilization is effective at reducing both vector populations and dengue infections in children [15]. The discussion outlined some of the challenges in implementing IVM at large scale such as establishing and sustaining inter-sectoral collaboration, and highlighted opportunities for advancing IVM for *Aedes* control.

Dr. Mylène Weill (Institute of Evolutionary Sciences, University of Montpellier, France), described the evolution of insecticide resistance in vectors of human diseases. Resistance to insecticides has spread in most mosquito vectors through selection. Different adaptive mechanisms have proliferated depending on the selective pressure and mosquito species. Non-synonymous mutations in insecticide targets such as AChE1 (*ace-1* mutation), GABA (*rdl* mutation) and the *para* voltage-gated sodium channel (*kdr* mutations) were compared in terms of their impacts on the fitness of mosquito populations [16]. For example, the G119S mutation conferring resistance to organophosphates and carbamates strongly impacts development time, mating competition, mortality, fecundity and predation in *Anopheles gambiae* and *Culex pipiens* [17, 18], though gene duplication serves to mitigate these fitness costs [19]. In *Ae. aegypti*, individuals possessing *kdr* mutations exhibited slower larval development and lower fecundity compared to their susceptible counterparts [20]. These trade-offs contribute to the persistence of insecticide resistance in the field, its local dynamics and spread among geographical areas. Understanding the relations between mutations, fitness and selective pressure in mosquito vectors of human diseases is needed to improve the management of resistance and contribute to the success of vector control.

During the ensuing general discussion, members of the audience raised concerns about the underestimated burden of arboviral diseases in Africa. It was proposed that screening for arbovirus serotypes in malaria negative cases could help to improve the surveillance and prevention of neglected arbovirus diseases in this part of the world. The WHO-TDR representative confirmed that the program will engage resources to improve the arbovirus diagnostic capacity of national centres in West Africa to prevent further outbreaks. The WHO-NTD representative also indicated that specific guidelines for the implementation, monitoring and evaluation of arbovirus vector control are being developed for the African region.

## Session 2: Insecticide resistance in arbovirus vectors

This session started with talks describing the status of insecticide resistance in arbovirus vectors in different regions of the world.

Dr. Beniamino Caputo (Sapienza University of Rome, Italy) presented data supporting the rise of pyrethroid resistance in *Ae. albopictus* in Italy, particularly in the northern regions where a chikungunya outbreak occurred in 2007. Insecticide resistance is also widespread in *Cx. pipiens* populations. In this area, the intense usage of chemical insecticides for urban mosquito control has likely exerted a strong selection pressure on *Ae. albopictus and Cx. pipiens.* Resistance management strategies should be further considered in Italy and more widely in Europe to contain insecticide resistance in arbovirus vectors.

Dr. Mamadou Coulibaly (University of Sciences, Techniques and Technologies, Bamako, Mali) reviewed the insecticide resistance status of *Ae. aegypti* and *Ae. albopictus* in Africa. Although these two vectors are present in several African countries, there is a paucity of data on insecticide resistance, probably due to the focus on malaria vectors. Available data showed that resistance of *Ae. aegypti* to DDT and pyrethroids is present in both central and West-African countries and involves both knock down (*kdr)* mutations and metabolic resistance mechanisms [21–26]. Resistance to other insecticide classes (organophosphates) has also been reported although data are limited to a few countries. In the context of the circulation of arboviral diseases in Africa, this presentation highlighted the need to improve the monitoring of insecticide resistance in *Aedes* mosquitoes in Africa, especially in Eastern regions from which data are absent.

Dr. Hassan Vatandoost (Tehran University of Medical Sciences, Iran) summarized the diversity of vector-borne diseases affecting the Eastern Mediterranean Region and their consequences for public health and then presented an overview of the resistance status of local populations of *Ae. aegypti* and *Ae. albopictus*. Data from this region support the presence of insecticide resistance although significant variations in resistance levels were recorded probably linked to different vector control practices and methods [27–29]. He concluded by emphasizing the need for better coordination between insecticide resistance monitoring and management at the regional level. He also reported some resistant status of other arboviral vectors such as *Culex*, Sandfly and ticks in the region.

Dr. Feng-Xia Meng (Chinese Center of Disease Control and Prevention, Beijing, China) started by describing the data management system for insecticide resistance in China. Vectors monitored include *Cx. pipiens*, *Cx. quinquefasciatus*, *Ae. aegypti*, *Ae. albopictus* and *Cx. tritaeniorhynchus*. She showed the results of insecticide resistance analyses indicating that *Ae. aegypti* and *Ae. albopictus* have developed resistance to pyrethroids in several provinces such as Guangdong and Yunnan whereas resistance to organophosphates and carbamates were moderate in most provinces. Both *kdr* mutations and metabolic resistance mechanisms have been detected in *Aedes* mosquitoes.

Dr. Kamaraju Rhagavendra (National Institute for Malaria Research, Delhi, India) described the diversity of arboviruses circulating in India and their consequences for public health. The few insecticide resistance data available revealed widespread resistance to DDT in *Ae. aegypti* across the country while resistance to organophosphates appeared more frequent in southern India. Pyrethroid resistance in *Ae. aegypti* and *Ae. albopictus* was reported in Delhi and Kerala regions [30–33]. Challenges for the control of arboviral diseases in India include the development of vector surveillance and resistance monitoring programs and the implementation of rational vector control strategies throughout the country.

Dr. Haroldo Bezerra (Pan American Health Organization, USA) presented the key challenges for strengthening vector management in the Americas. These include: evaluating the risk of arbovirus transmission, reducing mosquito breeding sites density, implementing efficient entomological surveillance and vector control, developing knowledge and skills in entomology, evaluating vector control practices and monitoring insecticide resistance. In the context of the increasing insecticide resistance of arbovirus vectors in the Americas, the PAHO promotes the implementation of integrated vector control (IVM).

Dr. Constância Ayres (Fundação Oswaldo Cruz, Brazil) presented an overview of the resistance status of *Ae. aegypti* in South America. She proposed that the eradication campaign implemented from 1950 to 1970 might have led to the selection and spread of DDT resistance. Decades later, the occurrence of temephos resistance due to the intensive use of organophosphates for larval control [34] has led to the gradual replacement of these insecticide families by *Bti* and insect growth regulators [35, 36]. Similarly, the use of pyrethroids for space spraying together with existing cross-resistance mechanisms (especially *kdr* mutations) previously selected by DDT led to the rapid rise of pyrethroid resistance. Overall, this presentation underscored the widespread high levels of insecticide resistance in South American *Ae. aegypti* that may impact the efficacy of conventional insecticides in the prevention of arbovirus transmission.

The following presentations were dedicated to the latest advances related to: the global mapping of insecticide resistance; the understanding of associated molecular mechanisms; the development of novel diagnostic tools; the evaluation of the impact of resistance on vector control; and the development of insecticide resistance management strategies.

**Fig. 3:**
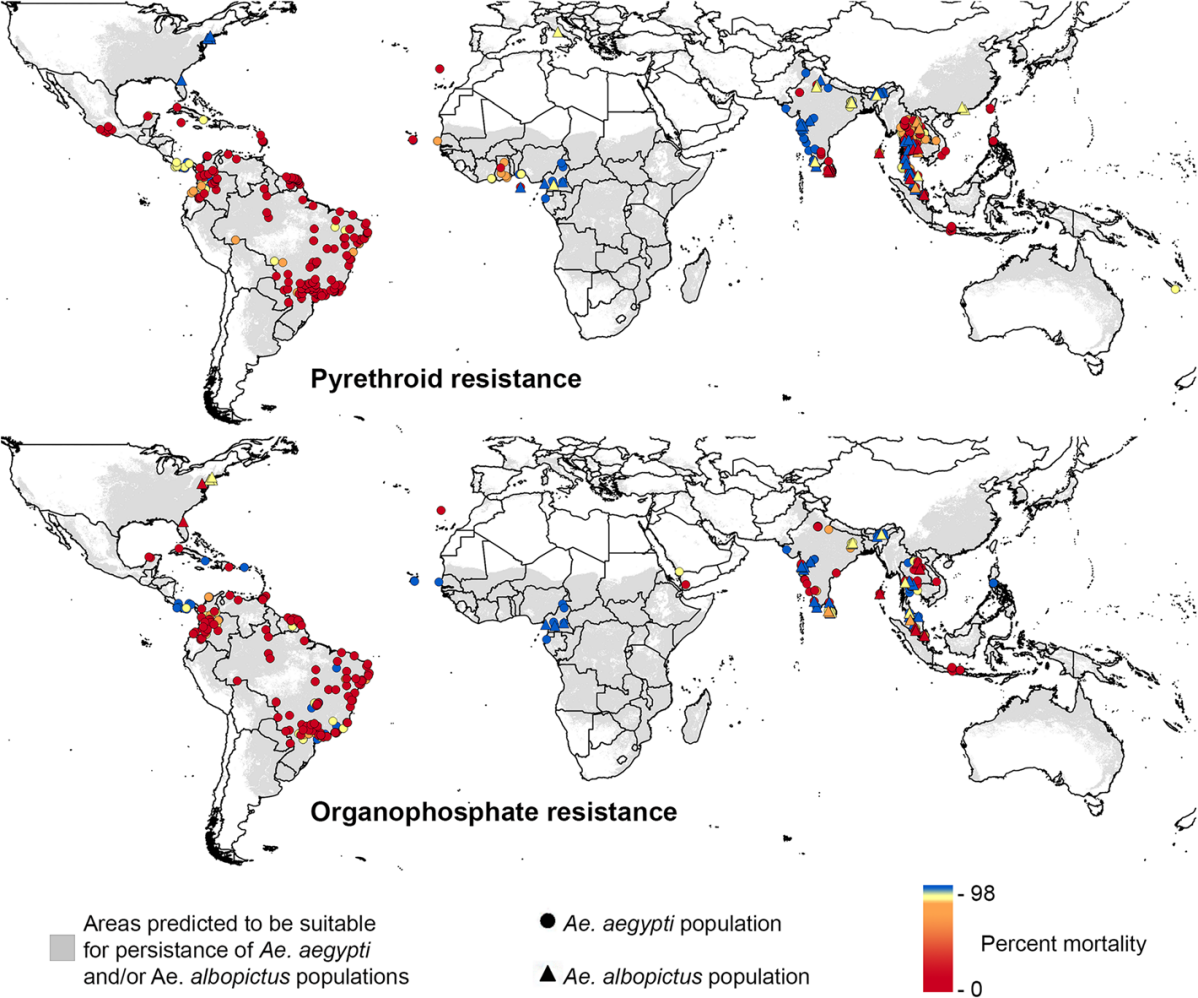
Global distribution of insecticide resistance data in *Aedes* mosquitoes

First, Dr. Catherine Moyes (University of Oxford, Oxford, UK) described the work performed by the WIN community to update insecticide resistance databases and generate worldwide resistance maps for *Ae. aegypti* and *Ae. albopictus.* About 6700 bioassay data points were collected from all continents and used for mapping the global distribution of insecticide resistance between 2008 and 2016. Overall, this work confirmed the wide distribution of insecticide resistance in *Ae. aegypti* to all insecticide families (Fig. 3). Although less information is available for *Ae. albopictus*, resistance to various insecticides was reported especially in Asia. Dr. Moyes presentation highlighted the need for standardized protocols and novel diagnostic tools for monitoring insecticide resistance worldwide.

Dr. Azael Che-Mendoza (National Center for Disease Control and Prevention, Ministry of Health, Mexico) described the main strategies that have been implemented by the Ministry of Health of México in response to insecticide resistance in the country. As observed in many places, the use of DDT in the 1950–1960s followed by the use of pyrethroids in the 1990s selected for *kdr*-based pyrethroid resistance in Mexico [37] that has significantly affected vector control efficacy. This in turn has led to changes in insecticide policy for mosquito control. The development of a nation-wide web-based surveillance systems integrating epidemiological, entomological and resistance data [38] aims to preserve the effectiveness of insecticides in the control of vector-borne diseases in Mexico.

**Fig. 4:**
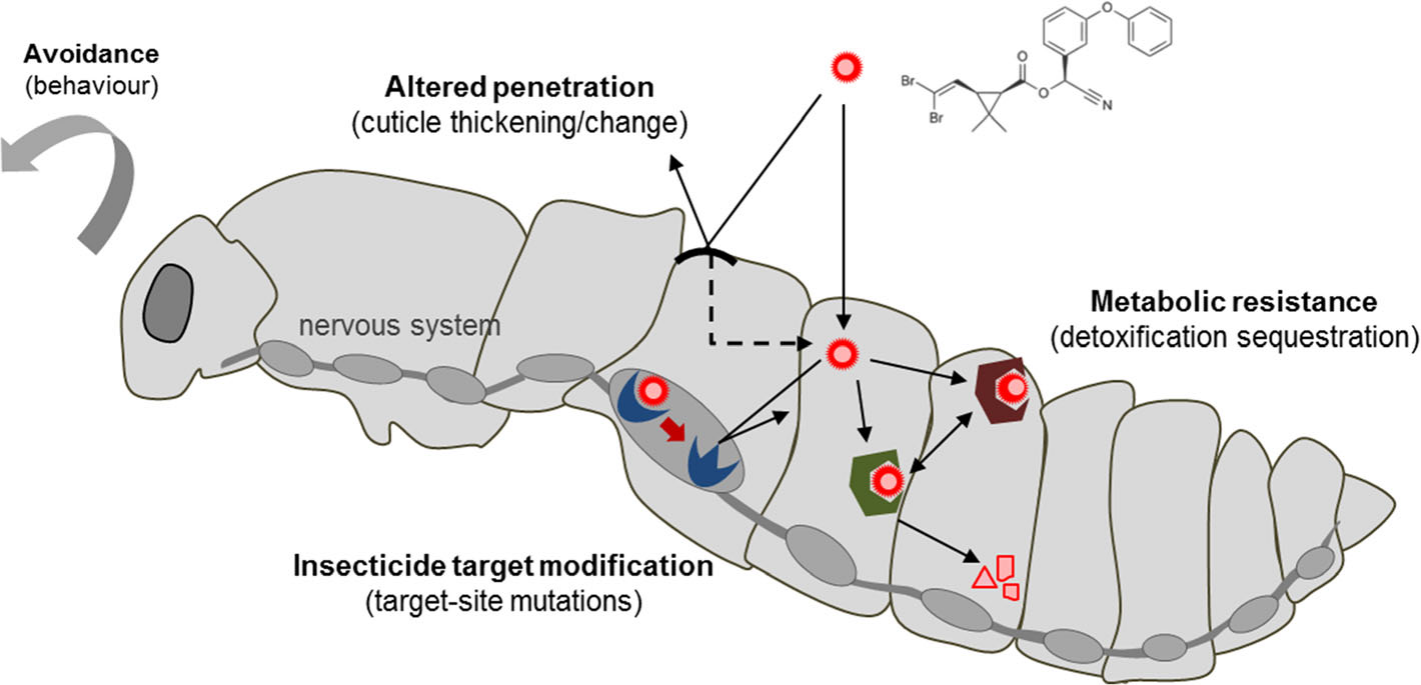
Mechanisms of resistance to chemical insecticides in mosquitoes

The following presentation by Dr. Shinji Kasai (National Institute of Infectious Diseases, Japan) focused on target-site mutations affecting the voltage-gated sodium channel targeted by pyrethroids and DDT, several of which confer *kdr* phenotypes (Fig. 4). Although multiple *kdr* mutations have been described in *Ae. aegypti*, electrophysiological studies suggest that only a few of them (i.e. S989P, F1534C, V1016G) appear to result in pyrethroid resistance phenotypes directly or synergistically, and that the combination of different mutations plays a key role in determining resistance levels to different pyrethroids [39]. The recent detection of mosquitoes carrying all three of these mutations in South-East Asia raised concerns for the management of pyrethroid resistance. In *Ae. albopictus*, individuals carrying mutations at the 1534 codon were recently detected in Singapore and China hence suggesting that the increasing exposure of the tiger mosquito to pyrethroids is selecting for resistance [40, 41]. Research priorities include studying the spatio-temporal dynamics of *kdr* mutations and validating their importance in the resistance phenotype to various insecticides [42].

Dr. Jean-Philippe David (Centre National de la Recherche Scientifique, Grenoble, France) gave a presentation about the genetics of metabolic resistance in arbovirus vectors (Fig. 4). After defining the evolutionary bases of metabolic resistance, the current knowledge on the role of detoxification enzymes in the resistance of *Aedes* mosquitoes to different insecticide families was reviewed and knowledge gaps identified. A global analysis of detoxification enzymes in multiple resistant populations from different continents identified a set of robust genes underpinning metabolic resistance in *Ae. aegypti* and *Ae. albopictus*. Recent genomic analyses suggest that copy number variations (CNV) play a key role in the over-production of detoxification enzymes conferring resistance to insecticides in *Aedes* mosquitoes, opening the way for the design of novel DNA-based diagnostic assays to track metabolic resistance in the field [43]. Future research efforts will aim to enlarge the panel of validated markers of metabolic resistance, evaluating their importance in conferring resistance to various insecticides, identifying their associated fitness costs and developing novel diagnostic tools for monitoring their dynamics in natural mosquito populations.

Dr. John Vontas (Foundation for Research and Technology - Hellas, Crete, Greece) presented the latest developments in diagnostic markers for tracking insecticide resistance in arbovirus vectors. He discussed the value of improving current resistance surveillance tools to guide decision-making process for resistance management. Dr. Vontas then reviewed resistance-monitoring tools such as bioassays, biochemical assays and molecular diagnostics tools both currently in use and those under development. Overall, this presentation highlighted the benefit of combining bioassays and molecular diagnostics for gathering contemporary and pertinent information for resistance management as well as for implementing integrated vector control approaches.

This session ended with a presentation from Dr. Isabelle Dusfour (Institut Pasteur de la Guyane, Cayenne, French Guiana) about insecticide resistance management strategies applicable to mosquito vectors of arboviruses. She emphasized that selection of resistance can be influenced by various factors including insect biology, genetics of resistance and insecticide applications. Experience from the past indicates that Insecticide Resistance Management (IRM) strategies must be integrated within vector control programs at an early stage before resistance occurs. IRM should include regular monitoring of resistance together with careful implementation, monitoring and evaluation of resistance-breaking interventions. Such interventions include rotations, mosaics or combinations of unrelated insecticides as well as source reduction. Developing insecticide resistance management plans for *Aedes* mosquitoes will require improving resistance monitoring systems, engaging populations to IRM, developing novel diagnostic tools, evaluating operational insecticide resistance risk levels, enlarging the panel of available insecticides, and promoting the use of non-insecticidal control tools as part of integrated vector control management.

## Session 3: Innovative vector control approaches for emerging arboviruses

**Fig. 5:**
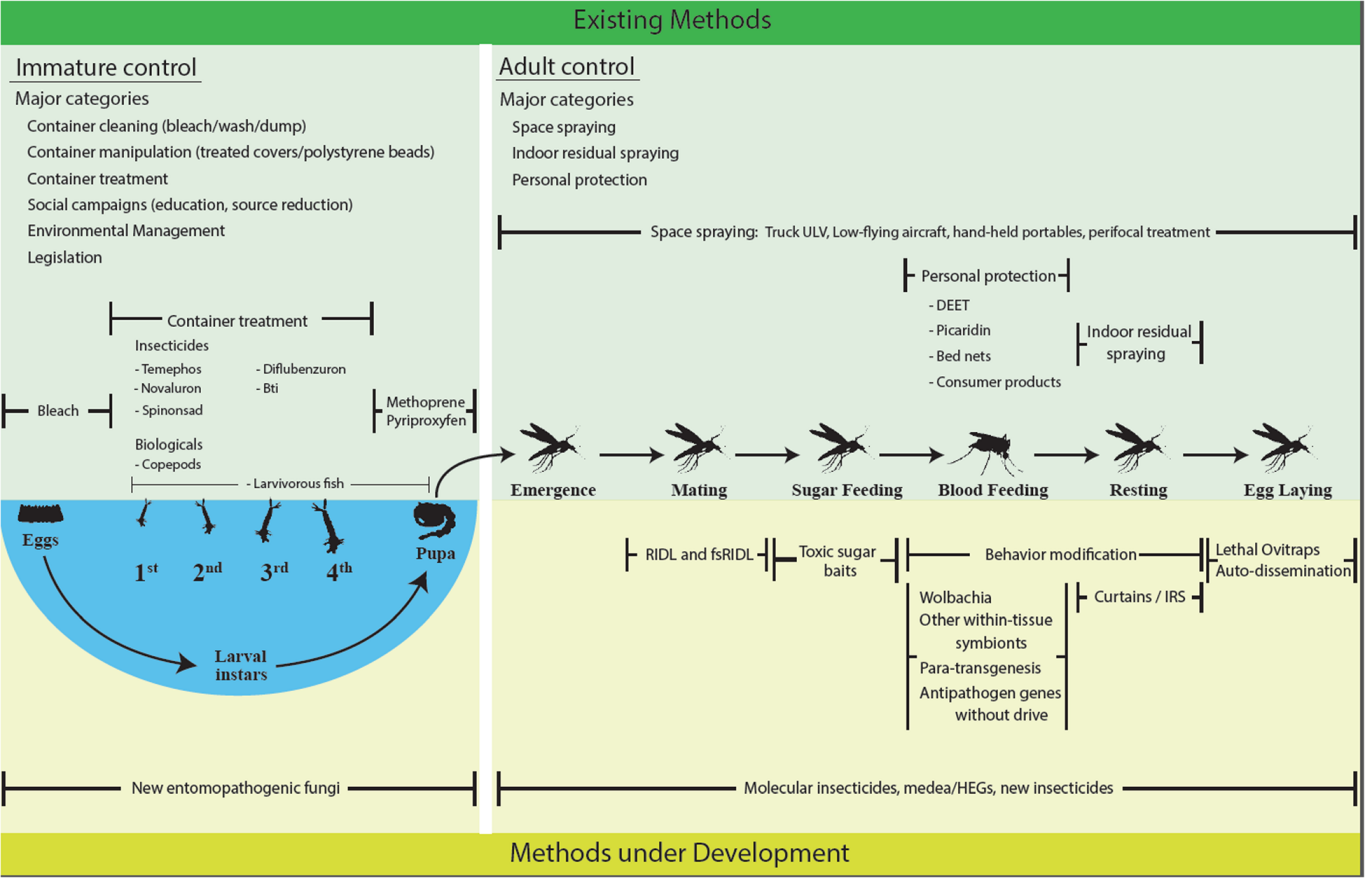
Some examples of new vector control tools having a potential role in insecticide resistance management in dengue vectors (Courtesy of Dr. N. Achee). Copyright: Creative Commons Attribution 4.0 License (https://creativecommons.org/licenses/by/4.0/). Citation: Achee et al. (2015) A critical assessment of vector control for dengue prevention. *PLoS Neglected Tropical Diseases* 2015; 9(5):e0003655 [68]

This morning plenary session opened with an introductory talk given by Dr. John Vontas (IMBB/Forth, Crete, Greece) in lieu of Dr. Nicole L. Achee (Eck Institute, USA) who was unable to attend the meeting. The talk contextualized the major obstacles that vector-borne disease control programs currently face in terms of the relatively limited methodological options available for vector control. Whilst current vector control methods can reduce transmission if rigorously applied, to date vector control has often been relatively ineffective in preventing the spread of arboviral infections. Coupled with the rise of insecticide resistance in *Aedes* populations and the limited arsenal of insecticides available for public health, this justifies the urgent need for new vector control tools. Mosquito ecology is the common denominator for the development of new vector control products [44] and over 16 novel approaches are in the pipeline targeting different phases of the mosquito’s life-cycle and specific behaviors (Fig. 5). Attractive toxic sugar baits, *Wolbachia*-based control, spatial repellents, self-limiting genetic technologies (e.g. SIT and RIDL), trapping methods, and new chemical insecticide-based approaches, including new products and mode of actions as well as auto-dissemination strategies were specifically described. Although promising, evidence-based demonstration of the epidemiological impact of most of these novel tools is missing and undermine calls for deployment at large scale.

Following the introductory note, the session had nine presentations focused on more specific developments in vector control. Three talks were specifically devoted to advances in insecticide-based vector control.

Dr. Fabrice Chandre (IRD, France) gave an overview of the potential use of mixtures and combinations of insecticides with different modes of action in the island of Martinique, where *Ae. aegypti* has developed resistance to organophosphate and pyrethroid insecticides [45]. Pilot studies have provided promising results both at the larval stage (spinosad + pyriproxyfen combination) and at the adult stage (neocotinoids + pyrethroid combination) [46, 47].

The use of synergists to overcome insecticide resistance was also the central topic of the presentation made by Dr. Bruno Lapied (Univ. Angers, France) who provided an overview of the recent advances in the development of alternative approaches using biological (insect viruses) and chemical (repellents) synergistic agents mixed with insecticides [48, 49].

Addressing personal protection needs, Mr. James Orsborne (LSHTM, UK) presented an update on the efforts to develop insecticide-treated clothing as a means of personal protection against *Aedes* bites and arbovirus transmission [50]. These studies involved both controlled testing in laboratory conditions (free flight rooms) and a school-based randomized trial implemented in Thailand enrolling children from ten schools with insecticide-treated school uniforms [51]. In the trial, treated clothing appeared to have reduced the *Aedes* population inside school in the first month of the study. However, due to poor insecticide retention within the clothing, there was no significant difference in dengue prevalence between insecticide-treated and control schools [52]. Novel treatment techniques and strategies were also presented. With innovative treatment techniques that bind deeper into fabrics increasing insecticide retention as well as development of ‘wash-in repellent detergents’ that could be rapidly distributed during an arbovirus outbreak.

Dr. Rui-De Xue (Anastasia Mosquito Control District, USA) gave a talk on new repellents for personal protection against arbovirus vectors. After reviewing formulations and efficacy data for commercially available repellents (e.g. DEET, picaridin, IR-3535), focus was given to some of the most promising new molecules that are currently being tested such as the experimental repellent piperidine. Oviposition repellents are also gaining importance in the treatment of container breeding sites.

Dr. Rui-De Xue also presented a talk on attractive toxic sugar baits (ATSB) for controlling arboviruses on behalf of Dr. Whitney Qualls (Texas Department of State Health Services, USA). This approach takes advantage of the sugar feeding behavior inherent to all adult mosquitoes. ATSB uses sugar as a bait to expose male and female mosquitoes to a deadly toxin (e.g. boric acid). The approach is also being tested in combination with pyriproxyfen and results point to effective control of both adult and larval *Ae. albopictus*, highlighting the versatility of ATSB for vector control [53].

**Fig. 6:**
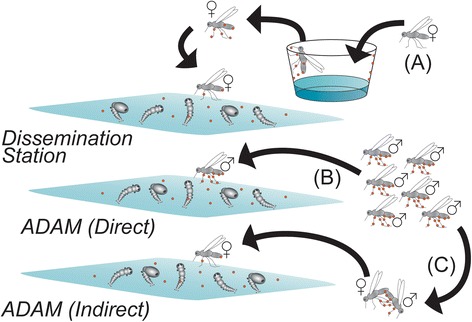
Diagram comparing the auto-dissemination station-based approach with the Auto-Dissemination Augmented by Males (ADAM) (Courtesy of Dr. S. Dobson). Copyright: Creative Commons Attribution 4.0 License (https://creativecommons.org/licenses/by/4.0/). Modified from the original picture by S. Dobson. Citation: Mains et al. (2015) Male mosquitoes as vehicles for insecticide. *PLoS Neglected Tropical Diseases* 2015, 9(1):e0003406 [55].

Dr. Stephen L. Dobson (University of Kentucky, USA) focused on autocidal methods for *Aedes* control. This strategy involves the use of mosquitoes as “insecticides-delivery” agents to reduce/suppress vector populations [54]. The talk summarized the results of Auto-Dissemination Augmented by Males (ADAM) strategy carried out in the USA [55]. The trial was based on the field-release of *Ae. albopictus* males contaminated with pyriproxifen (PPF). The males either contaminated the females during mating or directly contaminated larval habitats (Fig. 6). Although the *Ae. albopictus* population declined in one site following the introduction of PPF-treated males, further data on male dispersion and survival as well as on the size and location of the release are still needed to validate the efficacy of the ADAM strategy for *Aedes* control.

Dr. Margareth Capurro (Universidade de São Paulo, Brazil) gave a presentation on the recent developments towards the improvement of transgenic *Ae. aegypti* strains for the control of arbovirus transmission in Brazil. An overview of the ongoing experimental trials in Bahia, Brazil was given (e.g. PAT Project: open field release of OX513A *Ae. aegypti* transgenic line). The trial conducted over a year showed that the local *Ae. aegypti* population was reduced by 95% based on adult trap and 81% based on ovitrap indices compared to the adjacent no-release control area [56]. Experience suggests that genetic control tools may however be insufficient to completely suppress vector populations. An alternative would be to sequentially implement a suppression strategy to significantly reduce the mosquito populations followed by the implementation of a population replacement strategy. Such an approach should be implemented within the framework of integrated vector control.

Dr. Lee Ching Ng (National Environment Agency, Singapore) described the implementation of the “*Wolbachia* Project” in Singapore, for the control of *Ae. aegypti*. Following the recrudescence of dengue and emergence of Zika in Singapore [2], the National Environment Agency has studied the potential use of *Wolbachia*-infected males for population suppression. This ambitious project involved initial studies on the effectiveness of the *Wolbachia* population strategy strains, risk assessment analysis to ensure the safety of the technology and a strong community engagement component. A field calibration study was implemented to define the release strategy and the mosquito monitoring system (e.g. traps, sentinel sites, data analysis). The talk provided an overview of the developments of the project at different stages, highlighting the importance of community engagement as a means to increase receptivity to the vector control strategy.

The session ended with an open discussion between the speakers and the audience. The discussion highlighted the promising results so far obtained in the development of new vector control tools. However, more evidence-based data are required to assess the effectiveness and potential impact of novel tools especially if there are intended for use as part of IVM. Emphasis was made of the critical need for community engagement for the effective implementation of new vector control strategies. When suppression or replacement strategies have to be considered, it is highly recommended to release mosquitoes having “local genetic background” to avoid operational control failure (e.g. this was observed at Rio de Janeiro, Brazil during the first release of insecticide susceptible *Wolbachia*-infected mosquitoes that did not survive in presence of pyrethroids).

## Session 4: Private-public partnership for the development of new tools for arbovirus vector control

The session aimed at discussing the challenge of insecticide resistance in the context of developing new effective tools for insect vector control from the insecticide manufacturer’s perspective. Representatives of the agrochemical sector (28 companies were represented), Innovative vector Control Consortium (IVCC) and Insecticide Resistance Action Committee (IRAC) attended the workshop to present efficacy data and share their experience of vector control and resistance management.

Mr. John Lucas (Sumitomo Chemical Co., UK) provided an overview of the Insecticide Resistance Action Committee (IRAC) that was formed in 1984 to provide a coordinated industry approach to counter the development of resistance in pests and mites (http://www.irac-online.org/about/irac/). The challenge of insecticide resistance in insects that impact public health comes from the limited arsenal of new chemistries. This has been exacerbated by a major decline in the number of companies actively involved in insecticide development. Presently agricultural pesticides are being repurposed for vector control because vector control is a small market (2% of a total pesticide market estimated at over $50 billion USD), representing low profit and poor investment returns. Consequently the options for resistance management are few, and rely on appropriate resistance monitoring, proactive IRM strategies (e.g. mixtures, mosaic and sequences of unrelated insecticides) and susceptible genes preservation [57].

Mrs. Melinda Hadi (Vestergaard Frandsen, Switzerland) addressed the need for dedicated tools for monitoring insecticide resistance in *Aedes* mosquitoes. Vestergaard Frandsen has recently expanded IR mapper (www.irmapper.com), to include a mapping platform that provides geospatial displays of insecticide resistance data in *Ae. aegypti* and *Ae. albopictus* taken from peer-reviewed, published literature. The platform includes information on resistance to different classes of insecticides generated by different methods (e.g. test tubes and bottle assays, larval bioassays, biochemical and molecular tools). As of November 2016, 71% of the countries and territories reported confirmed resistance in *Aedes* spp. to at least one of the four main insecticide classes.

Dr. Julian Entwistle (Innovative Vector Control Consortium (IVCC), UK) provided a portfolio of new public health insecticide products under development funded by the IVCC. IVCC is a product development partnership that invests donor funds in R&D to overcome the financial barriers to product innovation in vector control, as described by John Lucas (http://www.ivcc.com/). Products that have been successfully developed so far include two long lasting indoor residual spraying products and two potentially-resistance breaking LLINs for malaria control. Three new paradigms targeting outdoor transmission are under consideration in Africa; attractive toxic sugar baits (ATSB), the push-pull strategy (spatial repellent with attractant baited traps) and swarm-targeted space sprays. Although the primary focus of IVCC has been on technology for the control of malaria vectors, many of the products and strategies that are being developed may be adapted to arbovirus vectors.

Dr. Frédéric Schmitt (Bayer, France) presented data on a new insecticide combination to control mosquitoes in areas of established or emerging resistance to conventional insecticides. The new formulated product for space spray application (Bayer708) combines two active ingredients with different modes of action i.e. a pyrethroid and a new AChR modulator belonging to the butenolid classes. Semi-field trials in USA and Brazil showed a very good efficacy of the combination against susceptible and pyrethroid resistant mosquitoes (*Aedes*, *Culex* and *Anopheles*) up to 100 m away from the spray source. In India, the combination sprayed indoors outperformed the local standard sprays (pyrethroid-based formulations) against *Cx. quinquefasciatus* and showed full efficacy against *Ae. aegypti*. The combination of two modes of action offers interesting potential for insecticide resistance management.

Mr. Kevin Riozzi (Yanco, UK) gave a talk on innovative insecticide products, including innovative mosquito coils and insecticide papers co-developed with Bayer Cropscience (Germany). According to the manufacturer, the insecticide paper containing 0.45% transfluthrin provides 100% fast kill (<10 min) against resistant mosquitoes indoors (*An. gambiae*, *Cx. quinquefasciatus* and *Ae. aegypti*). Insecticide papers offer interesting prospects for protecting vulnerable people against indoor and daytime mosquito bites at low cost.

Dr. Andreas Rose (Biogents AG, Germany) discussed the relevance of deploying combination of source reduction and traps for host-seeking females (BGS), or traps for gravid females (CDC-AGO & BG-GAT) to sustain vector control. Originally used in surveillance and monitoring, research has demonstrated the potential of BGS traps as a vector control tool [58]. Studies with area-wide use of AGO in Puerto Rico have shown an 80% reduction in the density of female *Ae. aegypti* up to 1 year [59] and significant reduction in chikungunya exposure [60]. In Brazil, a recent study showed significant reduction in abundance of gravid *Ae. aegypti* by BG-GAT [61]. According to the manufacturer, those traps are effective, practical, affordable, and could be easily deployed as part of IVM.

Mr. Yoshinori Shono (Sumitomo Chemical Co., Japan) presented the result of a new long lasting matrix-release formulation containing pyriproxyfen (SumiLarv®2MR) for larval control. Pyriproxyfen is an insect growth regulator with a low mammalian toxicity recommended by the WHO for use in drinking water [62]. According to the manufacturer, field simulated experiments showed that SumiLarv®2MR provided good efficacy against *Aedes* sp. up to 25 weeks after treatment. In Lao PDR, SumiLarv**®**2MR applied every 6 months to domestic water storage containers resulted in a significant reduction in *Ae. aegypti* larval densities for 18 months. The long-lasting efficacy of SumiLarv®2MR may allow reduction of the number of treatments per year, hence resulting in reduced operational costs.

Mr. Peter DeChant (ValentBioSciences, USA), presented resistance management in IVM programs based on operational use of *Bacillus thuringiensis israelensis* (AM65–52) for the control of dengue, chikungunya, and Zika vectors. Despite more than 32 years of operational use in mosquito control, no resistance has developed to commercial formulations of *Bti* AM65–52. The synergistic action of Cyt-1a with the 4 cry toxins offers a form of intrinsic resistance management, which protects against resistance development [63]. Direct application of Bti AM65–52 WG in Cambodia and Wide Area Spray of Bti AM65–52 WG in Malaysia reduced the incidence of human dengue from 43%–93%, respectively [64, 65]. The potential of *Bti* for IRM was discussed.

Dr. William Jany (Clarke International LLC, USA) presented the potential of using spinosad as a candidate for temephos resistance management in Brazil. Spinosad has a unique mode of action compared to other products by targeting nicotinic acetylcholine receptor (nAChR) subunits. Semi-field trials were conducted in 4 sites in Brazil using *Ae. aegypti* populations having different types of resistance. The final conclusion was that there was no cross resistance between temephos and spinosad and efficacy tests showed high residual activity of spinosad DT (mortality >80%) in treated tanks up to 8 weeks. The conclusion was that Spinosad may be an alternative tool for organophosphate resistance management.

**Mr. Herbert Nyberg** (New Mountain Innovations, USA) on the development of Acoustic Larvicide™ for Urban Mosquito Control. The principle is that specific acoustic resonance can induce the rupture of the dorsal tracheal trunk of mosquito larvae, causing death. According to the manufacturer, the Acoustic Larvicide™ is environmentally friendly, fast acting and highly specific. New Mountain Innovations has developed and in some cases already commercialized a wide range of acoustic products (ultra-portable, Remotely Operated Vehicle, etc.) to target various sizes and types of mosquito breeding habitats (e.g. small ponds, road ditch, sewer systems/natural, etc.). Mr. Nyberg reported that studies are ongoing to demonstrate the efficacy of Acoustic Larvicide™ to reduce mosquito population densities at the community level.

## Reports from round tables

### Round table 1: New tools for arbovirus vector control

#### New approaches to vector control

With insecticide resistance to existing Public Health insecticides increasing, control of adult *Aedes* will soon depend on a few insecticides with novel targets some still in development. Meanwhile there has been an increase in innovative strategies for the deployment of existing insecticides such as toxic sugar baits, insecticide treated paper, window curtains or eave access tubes, auto-dissemination traps, mass deployment of lethal ovitraps, and combinations of repellents and attractants (push-pull). New strategies for larval control are limited, but nonetheless include promising acoustic larvicides and entomopathogenic fungi. Furthermore, new approaches using the rickettsia symbiont, *Wolbachia*, or genetic modifications have revolutionized the sterile male technique (*Wolbachia* SIT and RIDL SIT), can also reduce larval production by interference competition (fsRIDL SIT) and create pathogen refractory adults (*w*Mel in *Ae. aegypti*). However, while these approaches are available and some already in use, they often require extensive and expensive multiple releases. This limitation can be addressed by the use of gene drive mechanisms that while promising are, however, still elusive and controversial.

#### Capacity building and new approaches to evaluation

Beyond new control approaches, there is a clear need for better equipment availability, standardization and calibration, trained personnel and funds to sustain high quality operations (i.e. capacity building). Fundamentally, however, there is a need to streamline registration of new products and for better epidemiological evidence to be incorporated into assessment standards. This may require the development of new strategies such as biomarkers of human vector exposure (e.g. antibodies or vector DNA) as part of the evaluation process paving the way for faster evaluation of effects on transmission risk, the ultimate metric of control effectiveness.

#### Development of new partnerships

Whilst recent suboptimal control by aerial spraying in Miami in response to the Zika threat implicate insecticide resistance (http://efish.fiu.edu/publications/Stoddard_Mosquito_spray_analysis_v3.pdf), in the USA and Europe the threat of resistance is perhaps less well acknowledged than in the tropics. This may be partly because in developed countries professionals not associated with research institutions often perform vector control and IR studies do not reach the peer-reviewed literature. Moving forward there is a clear need for better communication among researchers and Mosquito/Pest Control professionals to monitor, evaluate and minimize the development of insecticide resistance. Likewise there is a critical need to inform and collaborate with Public Health officials that excel in public outreach and, importantly, often control funding streams.

The following priorities were identified:

- Promote more bridges between Industry and Academia both to speed-up product development but also for a better evaluation of the relative effectiveness of different strategies for *Aedes* control and the development of protocols for post-application assessment (QA/QC) and epidemiological effects, as well as environmental side-effects.
- Development of an expert committee to evaluate prospective biocides and to define conditions for pilot deployment, especially in the context of combination interventions (IVM) to streamline registration of new products.
- Work on guidelines for good laboratory practice (GLP) and good experimental practice (GEP) with certified sites to speed up product evaluation and registration. Concomitantly, the development of a worldwide expert database would help in this way.
- Work on response plans adapted to countries need and capacities for arboviral diseases outbreaks, through optimized guidelines and making use of local resources (e.g. for countries with existing capacity for malaria control to co-opt resources, expertise and equipment for the control of vectors of arboviruses).
- Strengthen capacity of national authorities for vector control and particularly for testing and evaluation of insecticide products and new tools.

### Round table 2: Improving insecticide resistance surveillance and management

#### Diagnosis and interpretation of resistance

There is still a lack of accessible data sets documenting levels of insecticide resistance in countries affected by mosquito-transmitted arboviruses (especially in Africa and Australasia), and when the data are available, there is considerable variation in the methodologies used to record these data making cross-comparisons between sites very difficult. A lack of diagnostic doses for multiple insecticides was highlighted as a key problem, which, along with a lack of WHO impregnated paper supply for recommended concentrations, has undermined standardisation of resistance testing in *Aedes*. Other major obstacles relate to the interpretation of data, from an uncertainty in how to compare results from WHO tube bioassays and CDC bottle bioassays, to a more fundamental question of what results actually mean operationally (i.e. when should bioassay results lead to a change in vector control policy?).

Better facilities and capacity are required in most countries to implement programs for bioassay-based IR testing, with current resources often limiting activities to just one or two laboratories per country. Given current capacity limitations, and the need for some in depth dose–response data, a restricted sentinel site structure might represent a compromise, since spatial and temporal population heterogeneity in mosquito populations represents a challenge, and fine scale data will be required to calibrate predictive resistance maps.

Molecular diagnostics are currently underused for predictive purposes, and with DNA-based tests applicable to almost any samples, marker-based assays present great potential to yield fine-scaled data. Although pyrethroid and DDT target-site mutations are well known and can be detected by simple molecular diagnostic assays, genotype-phenotype association studies are needed to address the independent and combined effects of those mutations (for example, at least 10 mutations are associated with resistance phenotypes in *Ae. aegypti* but only a few have definitely been shown to lead to resistance). Functional validation of DNA markers for most metabolic resistance mechanisms is also a priority to speed up the implementation of resistance management strategies and help choosing alternative insecticide based on cross-resistance patterns associated with circulating resistance alleles.

#### Surveillance systems and cross-sectoral interactions

There are substantial gaps in surveillance systems for arboviral vectors, most notably in Africa facing increasing arbovirus outbreaks, and there is a general need for better cooperation among vector-borne disease control programmes targeting different diseases (such as arboviruses vs malaria), decision-making entities and funding agencies. A major problem has always been the poor organization and integration of resistance datasets generated by research institutions, as well as the interpretation and utilization of these data by relevant decision making entities. The effectiveness of cross-sectoral interactions also varies between countries; a problem that WHO through the Global Vector Control response (GVCR) will attempt to address *via* the establishment of national task force to make evidence-based recommendations on vector control, including how key interactors should work together (http://www.who.int/malaria/areas/vector_control/Draft-WHO-GVCR-2017-2030.pdf?ua=1&ua=1).

#### Insecticide resistance management (IRM)

This proved to be an area of considerable uncertainty, in part arising from difficulties underlying the interpretation of resistance bioassay data, but also in whether or how to integrate upcoming methods such as modified insect releases, which are immensely promising but for which costs, scalability and sustainability are unclear. Nevertheless, it is recognised that alternative or complementary control methods that do not rely on traditional neurotoxic insecticides, especially those with no recognized resistance in the wild (e.g. *Bti* or spinosad) have a major role to play. Fitness costs associated with insecticide resistance are also a crucial parameter in resistance management plans and require further exploration for both target-site and metabolic resistance.

A key consideration highlighted was the need for strong quality control at all levels of implementation, monitoring and evaluation of IRM programmes, without which success (or failure) cannot be judged. Helping low- and middle-income countries to strengthen their capacity to monitor insecticide resistance is a prerequisite to the implementation of IRM at global scale. The lack of global IRM plan for dengue and other arboviral diseases limits national capacities to support availability and accessibility of appropriate new vector control products.

The following priorities were identified:

- Establish insecticide resistance diagnostic doses for each vector species of arboviruses and faster supply delivery for insecticide testing to facilitate wider screening programmes.
- Determine the genotype-phenotype association and the predictive field value for target-site resistance markers (e.g. kdr mutations) to know which mutations are relevant for monitoring studies.
- Validation of metabolic-resistant markers and assays for metabolic mechanisms require further development.
- Encourage cross-sectoral interactions for improving insecticide resistance monitoring and management. Some examples of integration success stories (e.g. Sudan) and also particularly effective surveillance programmes (e.g. Mexico, Singapore) could serve as training examples for good practice.
- Consider the implementation of ‘resistance-free’ interventions wherever possible as alternatives to application of traditional neurotoxic pesticides. Develop evidence based guidelines to inform countries on how and when such interventions should be deployed at operational scale.
- When chemicals are in use for vector control, rotations, mixtures and/or sequences of molecules having different mode of action should be encouraged providing robust monitoring and evaluation of their resistance breaking potential.
- Development of a WHO GPIRM-style document for resistance management in vectors of arboviruses could be very valuable to give guidance on interpretation of data and recommended resultant actions.

## Conclusion: Toward the development of an international consortium for insecticide resistance surveillance and management in arboviruses vectors

Effective vector control has contributed to reducing arboviruses transmission worldwide and is an essential component of the WHO strategy for the prevention, control, and elimination of Neglected Tropical Diseases [66]. However, the last decades have seen emergence and expansion of insecticide resistance in vectors, coupled with a lack of appropriate response and preparedness for resistance management. The WIN workshop in Brazil highlighted the need for more bridges and partnerships between academia, research institutions, international organizations, stakeholders, the civil society and the private sector to manage insecticide resistance and sustain vector control in endemic areas and countries facing vector-borne diseases outbreaks.

In its first year of existence, the Worldwide Insecticide resistance Network (WIN) has demonstrated its potential for collating and interpreting data and enlisting cross-sectoral expertise in research and implementation on the control of vectors of arboviruses. The next step will be the development of an international consortium to lead research and training activities on insecticide resistance in vectors of emerging arboviruses and guide policies for vector control.
