# Erratum to: International workshop on insecticide resistance in vectors of arboviruses, December 2016, Rio de Janeiro, Brazil

**DOI:** 10.1186/s13071-017-2327-x

**Published:** 2017-08-21

**Authors:** Vincent Corbel, Dina M. Fonseca, David Weetman, João Pinto, Nicole L. Achee, Fabrice Chandre, Mamadou B. Coulibaly, Isabelle Dusfour, John Grieco, Waraporn Juntarajumnong, Audrey Lenhart, Ademir J. Martins, Catherine Moyes, Lee Ching Ng, Kamaraju Raghavendra, Hassan Vatandoost, John Vontas, Pie Muller, Shinji Kasai, Florence Fouque, Raman Velayudhan, Claire Durot, Jean-Philippe David

**Affiliations:** 1 Institut de Recherche pour le Développement (IRD), Maladies Infectieuses et Vecteurs, Ecologie, Génétique, Evolution et Contrôle (MIVEGEC, UM1-CNRS 5290-IRD 224), B.P. 64501, 911 Avenue Agropolis, 34394 Montpellier Cedex 5, France; 2 Center for Vector Biology, Rutgers University (RU), 180 Jones Avenue, New Brunswick, NJ 08901 USA; 3 Department of Vector Biology, Liverpool School of Tropical Medicine (LSTM), Pembroke Place, Liverpool, L35QA UK; 4 Global Health and Tropical Medicine, GHTM, Instituto de Higiene e Medicina Tropical, IHMT, Universidade Nova de Lisboa, UNL, Rua da Junqueira 100, 1349-008 Lisbon, Portugal; 5 Department of Biological Sciences, University of Notre Dame (UND), Eck Institute for Global Health, 239 Galvin Life Science Center, Notre Dame, Indiana, 46556 USA; 6 Malaria Research and Training Center (MRTC), Point G, Bamako, B.P. 1805 Mali; 7 Institut Pasteur de la Guyane (IPG), 23 avenue Pasteur B.P. 6010, 97306 Cayenne Cedex, French Guiana; 8 Department of Entomology, Kasetsart University (KU), 50 Ngam Wong Wan Rd, Ladyaow, Bangkok, Chatuchak 10900 Thailand; 9 Center for Global Health/Division of Parasitic Diseases and Malaria/Entomology Branch, U.S. Centers for Disease Control and Prevention (CDC), 1600 Clifton Rd. NE, MS G-49; Bldg. 23, Atlanta, GA 30329 USA; 10 Instituto Oswaldo Cruz (Fiocruz), Avenida Brasil 4365, Rio de Janeiro/RJ CEP, Manguinhos, 21040–360 Brazil; 11 Big Data Institute, Li Ka Shing Centre for Health Information and Discovery, University of Oxford, Oxford, OX3 7LF UK; 12 Environmental Health Institute (EHI), National Environment Agency (NEA), 11 Biopolis Way, Helios Block, #04-03/04 & #06-05, /08 Singapore, Republic of Singapore; 13 Department of Health Research, National Institute of Malaria Research (NIMR), GoI Sector 8, Dwarka, Delhi, 110 077 India; 14 Department of Medical Entomology & Vector Control, School of Public Health and Institute for Environmental Research, Tehran University of Medical Sciences (TUMS), Pour Sina Street, P.O. Box: 14155–6446, Tehran, Iran; 15 Institute Molecular Biology and Biotechnology (IMBB), Foundation for Research and Technology (FORTH), Panepistimioupoli, Voutes, 70013 Heraklio, Crete Greece; 16 Pesticide Science Laboratory, Agricultural University of Athens, Ieara Odoes 75, 118 Athens, Greece; 17 Department of Epidemiology and Public Health, Swiss Tropical and Public Health Institute, Socinstrasse 57, PO Box, 4002, Basel, Switzerland; 18 Department of Medical Entomology, National Institute of Infectious Diseases, 1-23-1 Toyama, Shinjukuku, Tokyo, Japan; 19 Vector Environment and Society Unit, The Special Programme for Research and Training in Tropical Diseases World Health Organization, 20, avenue Appia, CH-1211 Geneva 27, Switzerland; 20 Vector Ecology and Management, Department of Control of Neglected Tropical Diseases (HTM/NTD), World Health Organization, 20 Avenue Appia, CH-1211 Geneva 27, Switzerland; 21 Centre National de la Recherche Scientifique (CNRS), Laboratoire d‘Ecologie Alpine (LECA), UMR 5553 CNRS, 2233 rue de la piscine, 38041 Grenoble Cedex 9, France; 22 Université Grenoble-Alpes, Domaine universitaire de Saint-Martin d’Hères, 2233 rue de la piscine, 38041 Grenoble Cedex 9, France

## Erratum

After the publication of the article [1], it was realised that the attribution text for Fig. 3 was not correct. The corrected Fig. 3 attribution is included below.

**Fig. 3:**
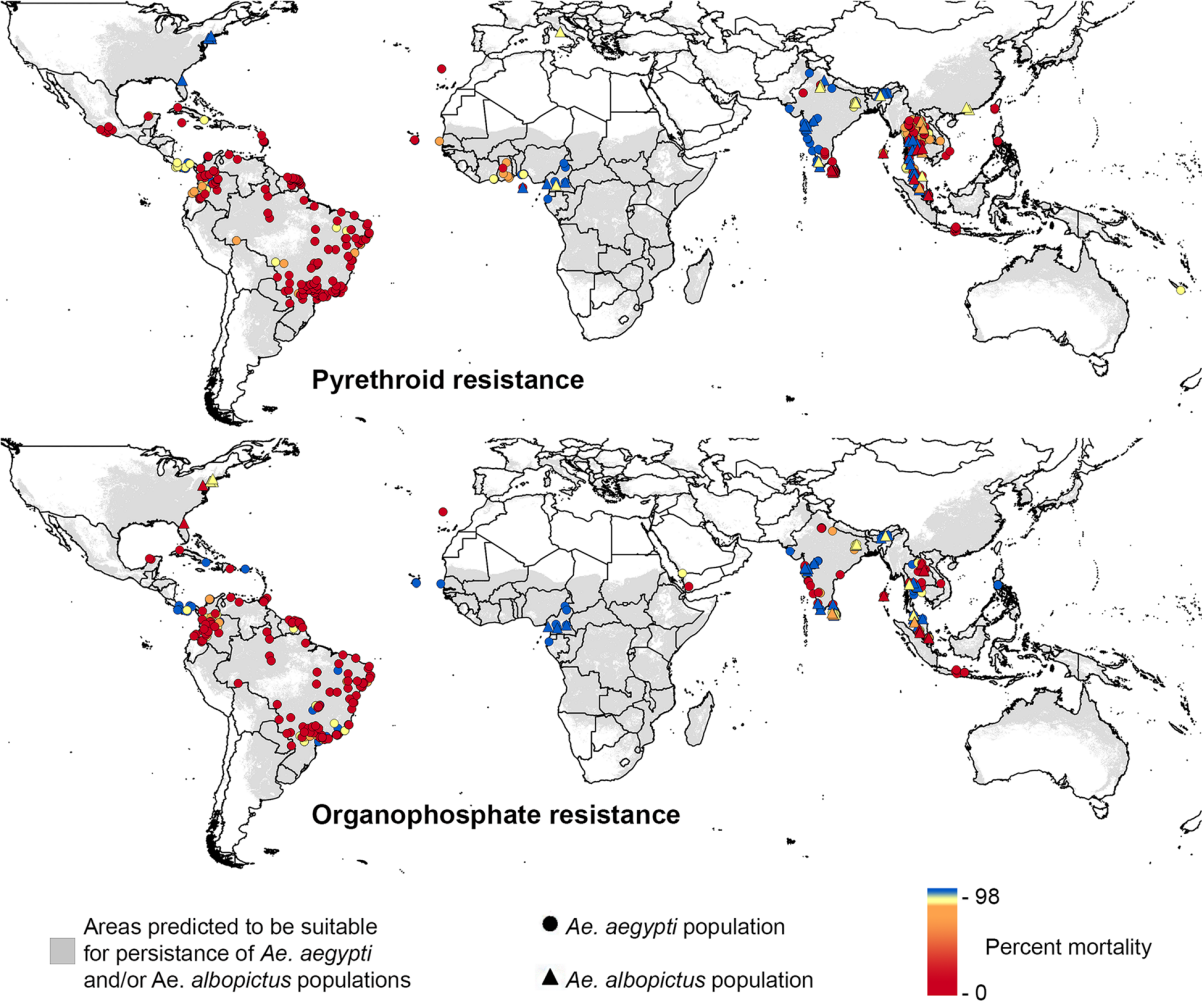
Global distribution of insecticide resistance data in *Aedes* mosquitoes. Insecticide resistance values from susceptibility bioassays are displayed on top of predicted areas of environmental suitability for *Ae. aegypti* and *Ae. albopictus*. The environmental suitability data layer shown was derived from Kraemer et al. [2]. Citation: Kraemer et al. (2015) The global distribution of the arbovirus vectors *Aedes aegypti* and *Ae. albopictus. eLife*. 2015;4:e08347 under the Creative Commons Attribute 3.0 Unported license (https://creativecommons.org/licenses/by/3.0/)

A corrected version of Fig. 3 is included with this Erratum.
